# Elf3 deficiency during zebrafish development alters extracellular matrix organization and disrupts tissue morphogenesis

**DOI:** 10.1371/journal.pone.0276255

**Published:** 2022-11-16

**Authors:** Swapnalee Sarmah, Matthew R. Hawkins, Priyadharshini Manikandan, Mark Farrell, James A. Marrs

**Affiliations:** Department of Biology, Indiana University-Purdue University Indianapolis, Indianapolis, IN, United States of America; Washington State University, UNITED STATES

## Abstract

E26 transformation specific (ETS) family transcription factors are expressed during embryogenesis and are involved in various cellular processes such as proliferation, migration, differentiation, angiogenesis, apoptosis, and survival of cellular lineages to ensure appropriate development. Dysregulated expression of many of the ETS family members is detected in different cancers. The human ELF3, a member of the ETS family of transcription factors, plays a role in the induction and progression of human cancers is well studied. However, little is known about the role of ELF3 in early development. Here, the zebrafish *elf3* was cloned, and its expression was analyzed during zebrafish development. Zebrafish *elf3* is maternally deposited. At different developmental stages, *elf3* expression was detected in different tissue, mainly neural tissues, endoderm-derived tissues, cartilage, heart, pronephric duct, blood vessels, and notochord. The expression levels were high at the tissue boundaries. Elf3 loss-of-function consequences were examined by using translation blocking antisense morpholino oligonucleotides, and effects were validated using CRISPR/Cas9 knockdown. Elf3-knockdown produced short and bent larvae with notochord, craniofacial cartilage, and fin defects. The extracellular matrix (ECM) in the fin and notochord was disorganized. Neural defects were also observed. Optic nerve fasciculation (bundling) and arborization in the optic tectum were defective in Elf3-morphants, and fragmentation of spinal motor neurons were evident. Dysregulation of genes encoding ECM proteins and matrix metalloprotease (MMP) and disorganization of ECM may play a role in the observed defects in Elf3 morphants. We conclude that zebrafish Elf3 is required for epidermal, mesenchymal, and neural tissue development.

## Introduction

Elf3 (also called E74 like ETS transcription factor 3, E74-like factor 3, ESX, ERT, ESE-1, EPR-1, JEN) is a member of the E26 transformation specific (ETS) family of transcription factors. ETS transcription factors share a highly conserved DNA binding domain, the ETS domain that possesses 85 to 90 amino acids rich in positively charged and aromatic residues. The ETS domain, which is localized at the C terminus of Elf3, forms three α -helixes and four-stranded β-sheets [1]. The α3 helix of the ETS domain interacts with the major groove of DNA; The 10 bp consensus sequence invariably contains the core motif GGA [2]. In addition to a functionally conserved ETS domain, Elf3 contains other regions required for its function: a SAM/pointed domain and a A/T Hook domain. The pointed domain is present within a subset of the ETS family and is involved in sequence specific DNA binding [3, 4]. A/T Hook domain binds to “AT” rich regions of DNA [5–7].

ELF3 has been reported to play significant roles in the development and progression of human cancers [2, 8–11]. ELF3 is implicated in breast cancer, thyroid cancer, colorectal cancer, lung cancers, oral cancer, pancreatic cancer, ovarian cancer, urothelial bladder carcinoma, prostate cancer, and synovial sarcomas [12–22]. A growing body of research shows that ELF3 can exhibit both tumor suppressor [16, 18, 23] and oncogenic functions in cancers [12, 14, 15, 22], a property seen in many transcription factors with dual biological function (both transcription activation and repression) [21, 24]. Deleterious loss of function mutations of ELF3 were documented and described as tumorigenic in many epithelial tumors [16, 18, 23, 24]. Conversely, ELF3 overexpression was also reported in other cancers including lung adenocarcinoma [12, 13, 15, 19, 24]. There is also evidence that ELF3 positively regulates TGF-βRII, which can inhibit tumorigenesis [11, 25, 26].

In addition to its role in oncogenesis, ELF3 regulates inflammation. ELF3 expression was reportedly restricted to epithelial tissues in normal conditions [5, 25, 27]. However, under inflammatory conditions, ELF3 becomes activated in various tissues and cell types [28–34]. Treatment with pro-inflammatory cytokines, interleukin-1β (IL-1β), and tumor necrosis factor-α (TNF-α) upregulated ELF3 expression in human bronchial airway epithelial cell lines [28–34]. Additionally, ELF3 expression was directly linked to increased expression of interleukin-6 (IL-6) in lung and serum of mice after induced pulmonary inflammation [26, 32]. The level of ELF3 protein was increased in osteoarthritic cartilages [35]. ELF3 acts as a regulator of cartilage catabolism and anabolism by acting as a transactivator for MMP13 transcription and as a repressor for collagen-2α1 (COL2α1) transcription in chondrocytes [35, 36].

ETS family transcription factors are involved in proliferation, migration, differentiation, angiogenesis, apoptosis and survival of cellular lineages during embryonic development [8, 37, 38]. However, little is known about the role of ELF3 in early embryo development. ELF3 regulates HLA-C expression on human placental extravillous trophoblast, which is involved in infection control and other complications in pregnancy [39]. In mice, *Elf3* expression increases after fertilization and remains high until the blastocyst stage [9]. *Elf3* knockout (*Elf3*-/-) caused lethality of about 30% of mice *in utero*. *Elf3*-deficent pups looked malnourished, lethargic, and had watery diarrhea [32, 40]. Those mice had defective small intestine: poorly developed villi, microvilli and abnormal morphogenesis and differentiation of enterocytes and mucus-secreting goblet cells [40]. ELF3 is involved in terminal differentiation of skin epidermis, epithelia of the cornea, keratinocyte and dendritic cell driven T cell differentiation in human [5, 32, 41–43]. Mouse mammary gland development and involution require *Elf3* [32, 44]. *Elf3* expression is also high in the retinal pigment epithelium (RPE) in mice [45]. These findings indicate that ELF3 is participates in various normal biological processes and disease conditions. However, its role in early development is poorly understood.

Here we report the distribution and loss-of-function consequences of Elf3 during zebrafish development. Morpholino oligonucleotide (MO) mediated Elf3-knockdown in embryos produced short and bent body, distorted fins, which show cartilage defects. Neural defects were seen in spinal motor neurons, brain and the visual system. Dysregulation and disorganization of extracellular matrix (ECM) components were observed. Our result show that Elf3 is required for epidermal, mesenchymal, and nervous tissue development, which may, in part, be due to defective ECM remodeling during embryogenesis.

## Materials and methods

### Zebrafish husbandry

Zebrafish (*Danio rerio*) AB strain was raised and maintained under standard laboratory conditions following Indiana University Policy on Animal Care and Use. IUPUI School of Science Institutional Animal Care and Use Committee approved the use of zebrafish adults for breeding, embryo collection and embryo experiments.

### Morpholino oligonucleotides and CRISPR/Cas9 gene targeting

Translation blocking antisense MOs (Gene Tools, Plymouth OR) were designed to target the *elf3* 5′UTR (***elf3-UTR-MO***: 5’ TGTGGTGGTCAAAGATTGTTTTCCA 3’) or the *elf3* translation start site (***elf3-ATG-MO***: 5’ TTAGACTAAGTTCGCTTGACGCCAT 3’). *elf3-UTR-MO* targets -36 to -12 nucleotides upstream of the start codon and *elf3-ATG-MO* targets 25 nucleotides of *elf3* coding sequence. The ENSEMBL transcript ENSDART00000113795.4/ elf3-201 (NCBI Reference Sequence: XM_002666100.3) was used to design the morpholino. A morpholino designed against human β globin pre-mRNA that targets the bases to correct a splice-generating mutation at position 705 was used as a standard control. It was shown that this morpholino has no target and no significant biological activity in zebrafish (Gene Tools). Independent control experiments were also performed using two independent guide RNAs to produce CRISPR/Cas9 knockdowns (S2 Fig in S1 File). To determine the optimum amount of each MO, 1–3 nL MO at different concentrations (0.01 μM–0.5 mM) were injected into 1–2 cell stage embryos. Concentration that consistently produced the phenotype without causing significant death of the embryos was used as the working MO concentration, which is 1 nL of 0.1 mM for each MO. Reproducibility and effectiveness were tested for each MO, showing that repeated experiments produced a consistent phenotype (both *elf3* MOs produced phenotypes shown here at similar amounts injected and at similar percentages, which could be reproduced in repeated experiments). Quantitative phenotype analysis is presented in Fig 2D (see below). In addition, CRISPR/Cas9 reagents (gRNA1: 5’ TGAACTACTGCACCATGGAT 3’; gRNA2: 5’ GAATCTGAACTACTGCACCA 3’) targeting *elf3* were injected into embryos (see supplementary materials and methods in S1 File), producing some embryos that showed similar phenotypes to the *elf3* MO injected embryos (bent body, small eye, small head, craniofacial defects). These *elf3* CRISPR targeted embryos showed evidence of insertions or deletions in *elf3* sequences after PCR amplification and sequencing their genomic DNA (see supplementary materials and methods in S1 File).

### Cloning of full length *elf3* cDNA

Total RNA was extracted from about twenty 2 days post fertilization (dpf) embryos using TRIzol, and cDNA was synthesized using 1 μg of total RNA, oligo(dT) primer and M-MLV reverse transcriptase (Promega). We amplified the open reading frame of *elf3* using 2 μL of cDNA, elf3-F: ATGGCGTCAAGCGAACTTAGTC and elf3-R: TTAATAACCGGTCTCCTCTATCC primers, Taq DNA polymerase with Thermopol Buffer (NEB). Gel electrophoresis showed the specific amplified product of the expected size (~1.1 kb), which was purified using Qiagen PCR purification kit. We cloned this cDNA into the pCBA3 expression vector, producing the plasmid *pCBA3-zf-elf3*. The cDNA was sequenced, which was submitted to the GenBank Database (OP605767).

To examine *elf3* expression during embryogenesis, total RNA was extracted from 4.5, 8, 20, and 24 hours postfertilization (hpf) embryos, and cDNA synthesis and PCR were done following the same procedures as described above.

### *In situ* hybridization and paraffin section

The plasmid *pCBA3-zf-elf3* was cut using Acc1 restriction enzyme and the *elf3* coding sequence 500 to 1128 bases were used to make the probe. A BLAST search ensured that there were no similarities between these *elf3* nucleotides and other zebrafish mRNA sequences. Digoxigenin labeled antisense probe was made using DIG RNA labeling kit and T7 RNA polymerase, and sense probe was used as a negative control, showing no staining. Whole-mount *in situ* hybridization of zebrafish embryos were performed as previously described [46]. Images were collected using a Leica MZ12 stereomicroscope.

*In situ* hybridization stained larvae were embedded in 1.5% agarose in 5% sucrose, and stored in 30% sucrose solution at 4°C overnight. Agarose embedded larvae were mounted with O.C.T. (Sakura Finetechnical Co.). Ten micrometer sections were cut using a Leica CM 3050 cryostat at −20°C and transferred onto Superfrost slides (Fisher). Images were acquired using a Zeiss Observer Z1 microscope (10X o.3 NA objective).

### Alcian blue staining

Alcian Blue staining was done as previously described [47]. Briefly, embryos from two independent experiments (~20 larvae per experimental condition per experiment) at 5 dpf were fixed in 4% phosphate-buffered paraformaldehyde (PFA), bleached in 10% H_2_O_2_ and 1 M KOH, and stained overnight in 0.1% Alcian Blue solution.

### Quantitative PCR analysis

Total RNA was extracted from 2 dpf embryos (~ 20 embryos) using the TRIzol (Sigma). One microgram of total RNA was reverse transcribed to cDNA using M-MLV reverse transcriptase (Promega) and oligo (dT) primer. The cDNA was diluted tenfold with RNase free water. Each target was amplified using 4 μL of cDNA, Power SYBR Green PCR mix (Applied Biosystems) and 0.5 μM of each primer. Ribosomal RNA *rsp15* was used as internal control. Primers used to quantify the expression of *mmp9*, *mmp13*, *mmp2*, *col2a1*, *Fn1*, *rsp15* genes and amplicon sizes are listed in S1 Table in S1 File. These primer sets were shown to produce a single product. Each reaction was performed in triplicate, and at least three independent mRNA samples for both conditions (control and morphant) were used for the experiment. Fold changes in gene expression were calculated using the comparative C_T_ method (ΔΔC_T_) as previously described [48].

### Immunohistochemistry

For whole-mount immunostaining, embryos were fixed in 4% PFA at 4°C overnight. Embryos were placed in blocking solution (PBS containing 0.5% Triton X-100, 1% DMSO, 5% goat serum, 1% bovine serum albumin, and 4% sodium azide) for 2 hours at room temperature. Primary antibodies against HuC/D (Sigma, 1:1000), acetylated tubulin (Sigma, 1:500), Col2α1 (Developmental Studies Hybridoma Bank 1:100), Fibronectin (Sigma, 1:400) were diluted in blocking solutions and incubated 1 to 2 days. Alexa Fluor 488-conjugated and Alexa Fluor 555-conjugated anti-mouse or anti-rabbit secondary antibodies (Molecular Probes, Grand Island, NY, USA) were used at 1:500 dilution. Nuclear staining was performed using TO-PRO-3 iodide at a 1:1000 dilution incubated for 1 hour at room temperature. Images were acquired using a Zeiss Observer Z1 LSM 700 confocal microscope (40X 1.1 NA W or 20X 0.8 NA objectives). Regions of interest in stained embryos/larvae were imaged with several confocal optical z-sections. Zen Black (Zeiss) software was used to produce maximum intensity image projections of the z-section volumes.

### Image analysis

Whole-mount acetylated tubulin immunostaining images of embryos were used to measure motor neuron axon lengths. Using ImageJ (free software from the National Institutes of Health, Bethesda MD), confocal images were calibrated to pixels per micron, and individual motor neurons in the anterior trunk were measured.

### Pathway analysis

Ingenuity pathway analysis (IPA) software was used to predict molecular interactions based on the manually curated content from human, mouse and rat publications in the Ingenuity knowledge base. “Experimentally observed or highly predicted” setting was used to predict potential Elf3 interactions with other molecules.

### Statistics

Unpaired two-tailed student’s *t*-test was used for comparisons between uninjected control and morpholino injected groups using GraphPad software (GraphPad Software, La Jolla, CA, USA).

## Results

### Expression of *elf3* during zebrafish embryogenesis

Previous studies in our laboratory showed that *elf3* was dysregulated by ethanol exposure during early development [46]. Experiments were performed to determine whether Elf3 controls developmental events that are affected by embryonic ethanol exposure, like craniofacial and brain development [49–52]. To determine the expression of *elf3* during zebrafish development, *elf3* gene sequences were amplified and cloned and sequenced. Zebrafish Elf3 and the human ELF3 protein share 47.31% identity, and ETS domain of zebrafish Elf3 is highly conserved, sharing 91.76% identity with human ELF3 ETS domain (S1 Fig in S1 File).

**Fig 1:**
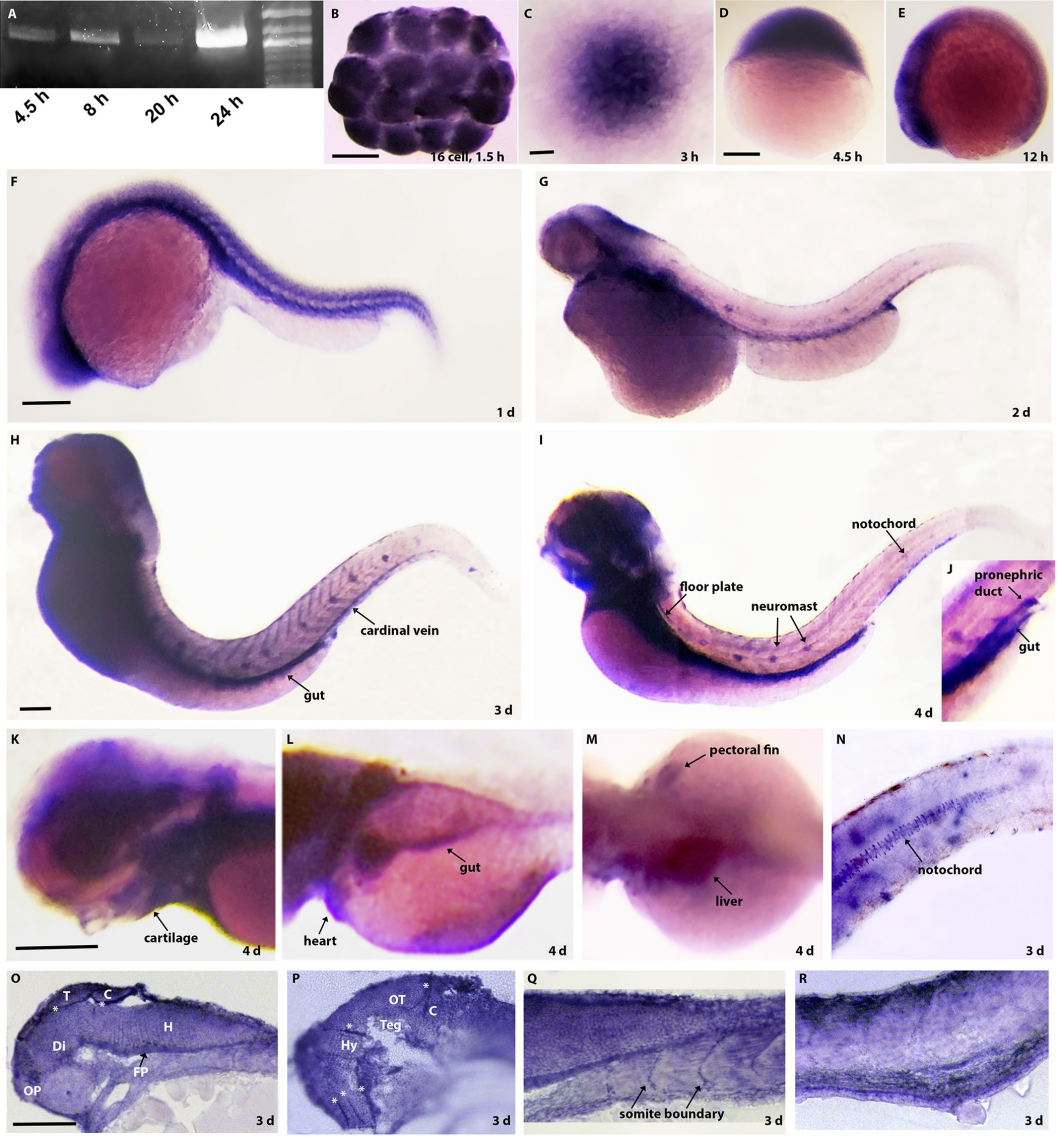
Expression of *elf3* mRNA during zebrafish development. (A) PCR detects *elf3* mRNA during blastula (4.5 hpf), gastrula (8 hpf), and somitogenesis (20 and 24 hpf) stages. (B) *In situ* hybridization analysis shows *elf3* mRNA expression in the 16 cell-stage embryos confirming maternal deposition. (C-D) During cleavage and blastula stages, *elf3* mRNA is ubiquitously expressed. (E) During early somitogenesis period, *elf3* mRNA is expressed more in the anterior region compared to the posterior region. (D-I) *In situ* hybridization detecting *elf3* expression in 1 to 4 dpf larvae shows mRNA expression in distinct tissues. (J-N) High magnification images of 4 dpf stained larvae show *elf3* mRNA expression in gut, pronephric duct (J), eye, cartilage (K), gut, heart (L), liver, pectoral fin (M), and in notochord (N). (O-R) 10 μM cryosections of the stained 3 dpf larvae show *elf3* mRNA expression in different brain tissues and higher expressions in the boundaries of those tissues (O, P), in somite boundaries (Q) and in the gut and pronephric duct (R). T: telencephalon; C: cerebellum; OP: olfactory pit; Di: diencephalon; FP: floor plate; H: hindbrain; OT: optic tectum; Teg: tegmentum, Hy: hypothalamus; white asterisk: tissue boundary. Scale bar for B; C; D-E; F-G; H-I; K-M; and O-R = 200 μm.

To determine the temporal and spatial expression patterns of *elf3* during zebrafish development, we analyzed mRNA by performing RT-PCR and *in situ* hybridization at various developmental stages. RT-PCR detected *elf3* transcript at 4.5, 8, 20, and 24 hpf embryos (Fig 1A). *In situ* hybridization detected *elf3* mRNA at 1.5 hpf (16 cell; prior to midblastula transtion) embryos, indicating maternal deposition (Fig 1B). *In situ* hybridization also showed *elf3* mRNA enriched cells at 3 hpf, being uniformly distributed in the blastula stage embryo. During somitogenesis, *elf3* mRNA was highly expressed in the forebrain, midbrain, midbrain-hindbrain boundary, and hindbrain, and relatively low levels of expression were detected in the posterior region and in the notochord (Fig 1C–1E). At 24 hpf, high *elf3* mRNA expression was detected in the eye primordia, developing liver, pancreas, blood vessels, intersomatic boundaries, and CNS regions (Fig 1F). Whole mount preparations of 2 to 4 dpf larvae showed that the expression *elf3* was more enriched in olfactory pit, eye, midbrain, midbrain-hindbrain boundary, craniofacial cartilages, liver, pancreas, gut, pronephros, neural tube, vacuolated cells in notochord, neuromast, pectoral fin, heart, cardinal vein, and caudal fin later during development (Fig 1G–1N). Sections of the 3 dpf stained larvae show *elf3* expression in brain regions and, interestingly, very strong expression at the boundaries of brain regions (Fig 1O and 1P) and somite boundaries (Fig 1Q and 1R).

### Knockdown of Elf3 severely affected zebrafish morphogenesis

To determine the function of Elf3 during development, we perform Elf3 loss-of-function experiments by injecting 1 to 2 cell embryos with antisense morpholino oligonucleotide (*elf3-UTR-MO* or *elf3-ATG-MO*) designed to block translation of *elf3* mRNA. As a negative control, an equal amount of the morpholino designed against human β globin pre-mRNA was also injected into 1 to 2 cell embryos. Either *elf3-UTR-MO* or *elf3-ATG-MO* morpholino injected embryos had no significant effects on epiboly progression. At 8.3 hpf, uninjected control embryos reached 82% epiboly (81.7 ± 4.39%; n = 41); control morpholino injected embryos reached approximately 85% (85.2% ± 4.19; n = 54, standard deviation); and the *elf3-ATG-MO* morpholino injected embryos reached approximately 76% epiboly (75.9% ± 6.48; n = 68).

**Fig 2:**
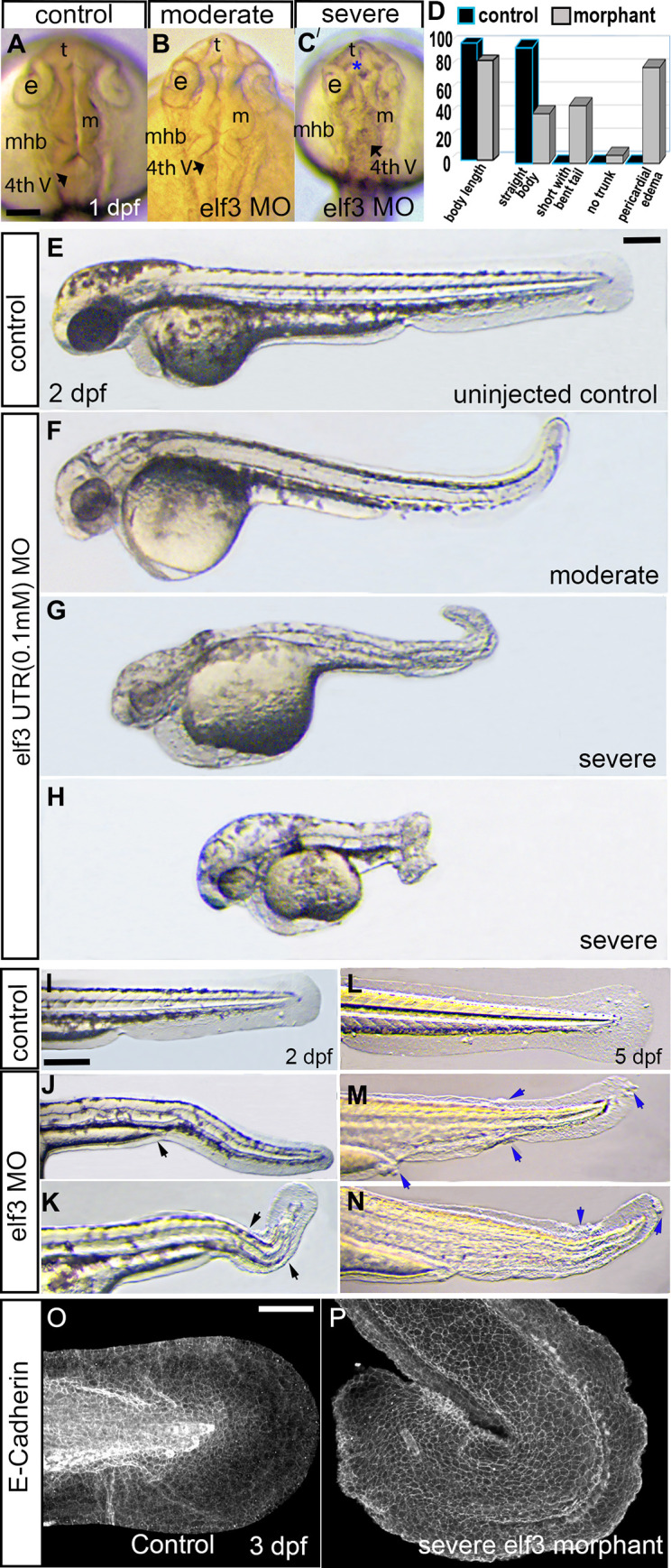
Knockdown of Elf3 severely affected zebrafish development. (A-C) Live images of uninjected and *elf3* morpholino injected embryos show development of the eye cup, telencephalon (t), forebrain ventricle, mesencephalon (m), midbrain-hindbrain boundary (mhb), and fourth ventricle in 1 dpf control (A) and moderately affected (B) and severely affected (C) embryos. Blue asterisk in C: dead cells. (D) First two bar graphs represent the body length percent of the control (black bar) and the morphant (grey bar) embryos (control embryo:100% ±4.5 (n = 39); *elf3-UTR-MO* injected embryo: 85.16% ±11.35 (n = 65; p<0.001). Other bar graphs represent the percent of the control and the morphant embryos with the labeled phenotype. (E-H) Live images of 2 dpf uninjected and *elf3* morpholino injected embryos show normal morphology of the control embryo (E) and short body, bent tail, small eye, pericardial edema, twisted notochord, and aberrant fin-folds of the morphant embryos (F-H). (I-N) Live images show normal median and caudal fin-fold in control (I, L), and irregular or absence of fin-fold (black arrows) and lumps in the fin fold (blue arrows) in morphants at 2 and 5 dpf. (O, P) 3D reconstruction of the confocal images of the E-cadherin antibody stained larvae show regularly-shaped fin edge of the control larvae and jagged fin-edges of the morphants. Scale bar for A-C; E-H; and I-N = 200 μm. Scale bar for O, P = 100 μm.

At 24 hpf, the *elf3* MO injected embryos had mild to severe brain morphogenesis defects, including abnormal midbrain-hindbrain boundary formation. Those embryos displayed delayed optic cup development or smaller optic cup (Fig 2A–2C). Severely defective embryos had dead cells in the forebrain ventricle region (Fig 2C). Control MO injected embryos had no defects (S2A, S2B Fig in S1 File). A positive control experiment was done by CRISPR/Cas9 knockdown using 2 independent guide RNAs targeting *elf3*. Injection of CRISPR/Cas9 reagents produced similar phenotypes as MO injected larvae (S2C-S2G Fig in S1 File). Sequencing genomic DNA of those larvae showed altered sequences of the targeted region suggesting disruption of Elf3 (S2C”-S2G” Fig in S1 File). Body length was measured at 2 dpf, which showed that the Elf3 morphants were shorter than the control embryos (control embryo:100% ±4.5 (n = 39); *elf3-UTR-MO* injected embryo: 85.16% ±11.35 (n = 65; p<0.001) (Fig 2D).

The Elf3 morphants were grouped as mild-, moderate- and severe-morphant groups. The mildly affected morphants (~43% of the total morphant embryos) were slightly shorter than the uninjected control but otherwise looked similar to control. The moderately affected morphant embryos (~50%) had a short body with a bent tail, pericardial edema, small eye, distorted notochord, aberrant/almost absent fin-folds, and reduced pigmentation (Fig 2D–2H). Severely affected morphants (~ 7%) had either a very short “pig” tail or almost no tail in addition to the defects described above present in moderately affected morphants (Fig 2G and 2H). Tail and fin fold defects became more severe later in the larval stages. Median and caudal fin fold edges were irregular and the morphant larvae often had cyst or clumps of cells in the fin fold (Fig 2M and 2N). The 5 dpf morphants showed structural alteration of actinotrichia fibers, the brush-shaped collagenous structures in the caudal fin while the control larvae had radial symmetrical arrangement of the actinotrichia fibers (Fig 2L–2N). Some larvae displayed almost collapsed fin folds (Fig 2K–2N). Labeling of fin fold epidermal cells by E-cadherin (Cdh1) antibody and 3D imaging confirmed severely distorted fin folds of the morphants (Fig 2O and 2P).

**Fig 3:**
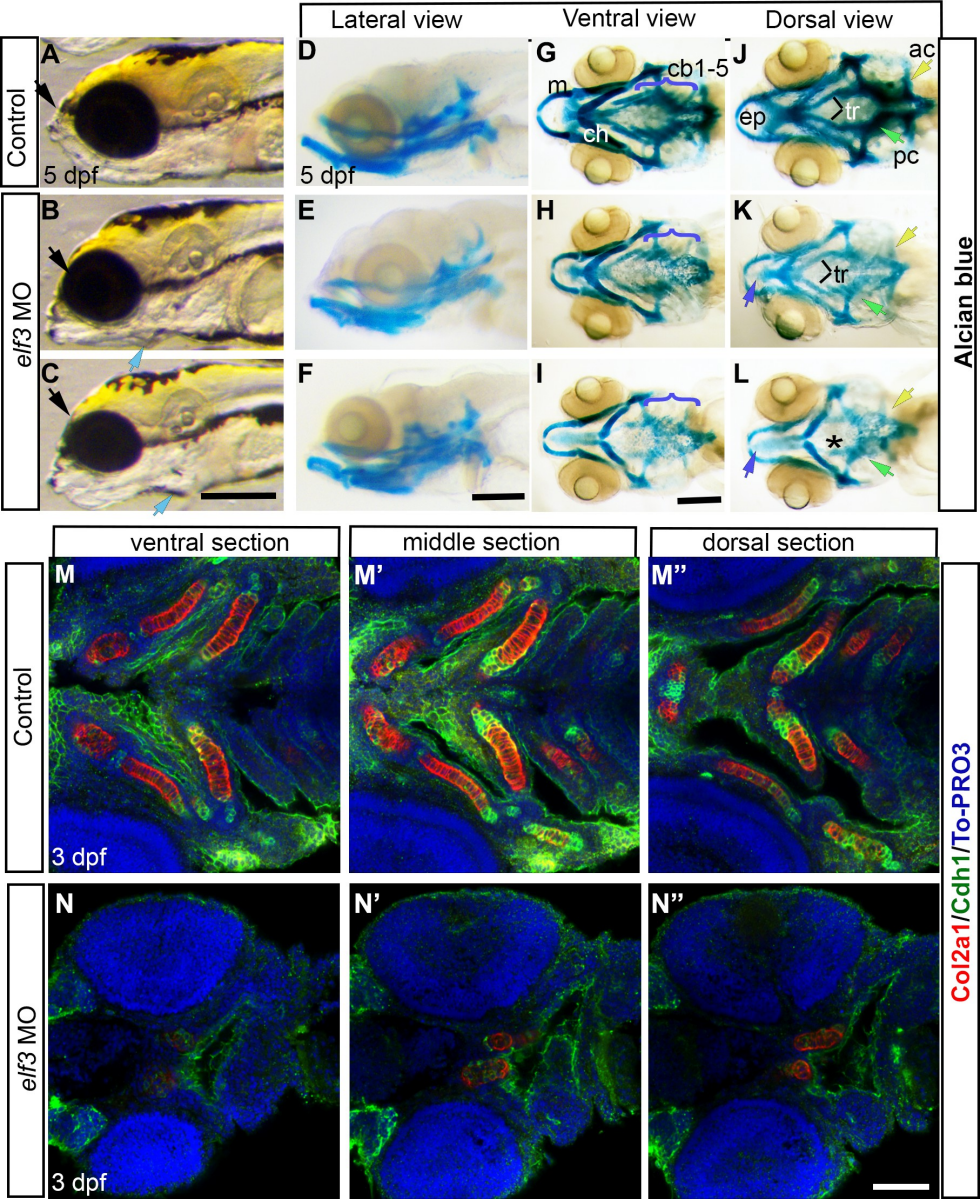
Elf3 knockdown affected craniofacial development. (A-C) Live images of 5 dpf Elf3 morphants show protruded jaw (blue arrow) and smaller head (black arrow) and eye (B, C) compared to control (A). (D-L) Alcian blue stained head skeleton of control and morphant larvae. Ventral view shows normal cartilages in control (G) and near-normal Meckel’s (m) and ceratohyal cartilages (ch) and poorly developed branchial cartilages (cb 1–5) in morphants (H, I). Dorsal view shows normal neurocranium in control (J) and smaller ethmoid plate (purple arrow), lack of trabecula (tr and *), parachordal cartilage (pc and green arrow) and auditory cartilage (ac and yellow arrow) in morphants (K, L). (M-N”) Col2α1 and E-cadherin (Cdh1) antibody stained 3 dpf larvae marked developing Meckel’s, Ceratohyal, 1^st^ and 2^nd^ branchial arches, and ethmoid plate in control larvae (M-M”) and a pair of cartilage (presumably Ceratohyal) in the morphant larvae (N-N”). Scale bar for A-C; D-F; and G-L = 200 μm. Scale bar for M-N” = 100 μm.

Anterior regions of the control and morphant larvae were compared. Morphant larvae had smaller heads compared to control. Smaller forebrain was evident in 5 dpf larvae (Fig 3A–3C). The morphants also showed jaw defects at 5 dpf (Fig 3A–3C).

### Deficiency of Elf3 during development causes malformation of craniofacial cartilages

To examine the craniofacial development in the morphants, Alcian blue staining was performed. Alcian blue-stained 5 dpf Elf3 morphant larvae showed relatively normal Meckel’s and ceratohyal cartilages but ceratobranchial arches (1–5) are either lightly stained or not stained, indicating poorly developed branchial cartilages with less proteoglycans in the extracellular matrix. Neurocranium development was also severely affected in Elf3 morphants, lacking the lateral regions of the ethmoid plate, showing deformed or missing trabecula, parachordal cartilage and auditory cartilage. Compromised craniofacial cartilage development was also observed in CRISPR/Cas9 injected larvae (S2C’-S2G’ Fig in S1 File).

In zebrafish, cranial chondrogenesis begins at 2 dpf when migratory neural crest cells arrive at the destination and aggregates into pre-cartilage condensations. These condensations are associated with increased cell adhesion and formation of gap junctions [53]. Cartilage morphogenesis occur by re-organization of the chondroprogenitors, followed by a growth phase. Growth is robust near the joints between cartilages. Chondroprogenitors matures and differentiate into chondrocytes, which is characterized by the deposition of cartilage matrix containing collagen II, IX, and XI and aggrecan [54].

To examine the developing cartilage and chondrocyte differentiation in Elf3 morphant, 2 and 3 dpf larvae were stained with collagen type II alpha-1a (Col2α1) antibody. Pairs of trabecula cartilages with Col2α1 positive chondrocytes were detected in control embryos at 2 dpf. At 3 dpf, Col2α1 was detected in developing Meckel’s, ceratohyal cartilage, 1^st^ and 2^nd^ branchial arches, and ethmoid plate in control larvae (Fig 3M–3M”, S3A–S3A", S3E-S3E” Fig in S1 File). However, Col2α1 expressing cells were dramatically reduced in Elf3 morphant at 3 dpf. Only a few cartilage cells, in what appeared to be trabecula cartilages, had Col2α1 expression (Fig 3N–3N”, S3B–S3B”, S3F–S3F” Fig in S1 File). Immunostaining using anti-E-cadherin (Cdh1) antibody detected high cadherin expression near the growing ends of cartilages of un-injected control larvae (Fig 3M–3M”, S3C–S3C”, S3E’–S3E” Fig in S1 File). Cadherin expression was also considerably reduced in the developing cartilages of the Elf3 morphant larvae (Fig 3N–3N”, S3D–S3D”, S3F–S3F” Fig in S1 File).

### Knockdown of Elf3 disrupts the expression pattern of Col2α1 in the fin folds and notochord

**Fig 4:**
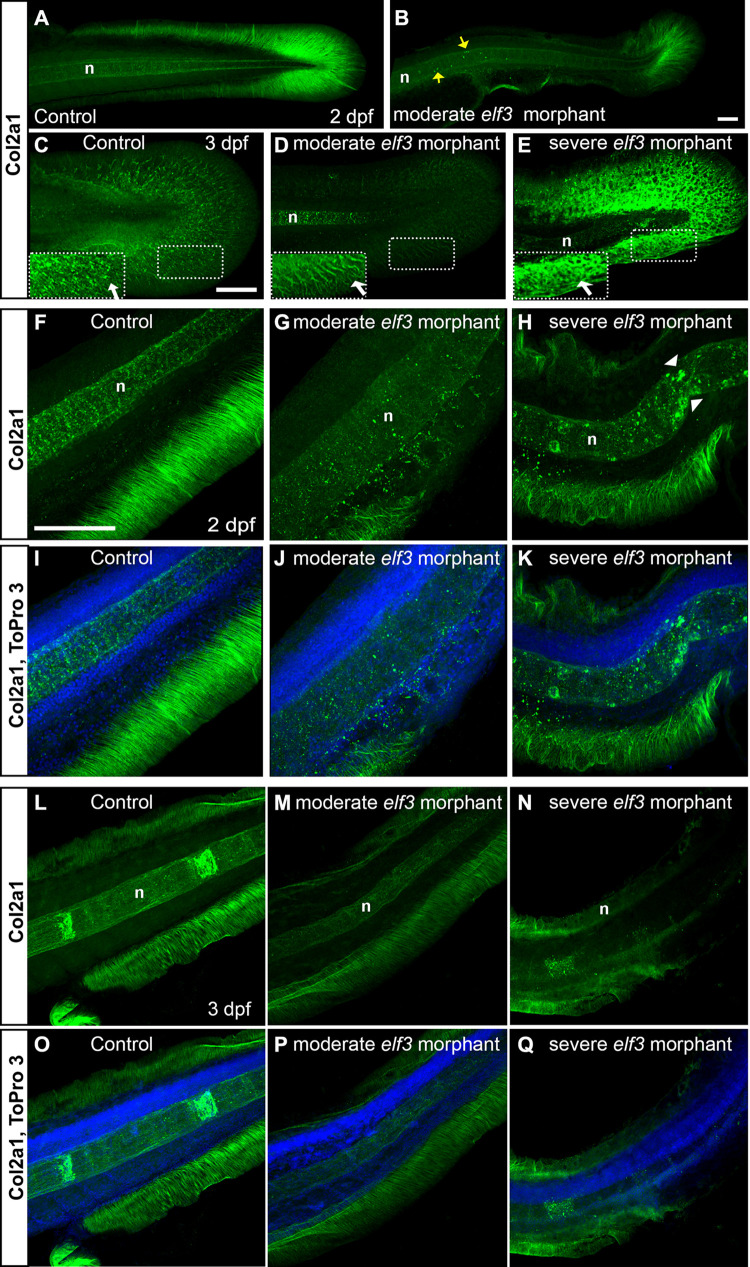
Elf3 deficiency led to disruption of Col2α1 expression in the fin and notochord. (A-E) 3D reconstruction of the confocal images of the Col2α1 antibody stained embryos show dense Col2α1 fibrils radially distributed throughout the whole caudal fin in the control (A, C) and less dense, irregularly distributed fibrils in moderately affected morphants (B, D); severely affected morphant showed abnormal accumulation of Col2α1, but no fibrillary pattern (E). Insets show high magnification of the marked rectangle area. yellow arrow: abnormal Col2α1 accumulation within notochord; white arrow: Col2α1 fibrils. (F-Q) Optical section of the confocal images of the Col2α1 antibody stained embryos show severely disrupted and disorganization median fin fold collagen fibrils in the *elf3* morphants at 2 dpf (G, H, J, K) and 3 dpf larva (M, N, P, Q) and nicely organized, thick fibrils in the control (F, I, L, O). Scale bar for A, B = 50 μm; for C-E = 100 μm; and for F-Q = 100 μm.

Proper cell-cell and cell-matrix interactions are essential for tissue formation. Epidermal cell shape changes and correct ECM assembly are essential steps in zebrafish median fin fold and caudal fin morphogenesis [55, 56]. These tightly regulated processes lay the foundation for subsequent steps of fin development [55]. Fin ray expansion occurs by the assembly of actinotrichia and lepidotrichia at the basal membrane of epidermal cells [56–58]. Collagens like Col2a1 and Col1a1 are essential for actinotrichia formation [56, 59–61]. Severely deformed actinotrichia of Elf3 morphants prompted us to examine the expression of Col2α1 in the median fin fold and actinotrichia. Col2α1 was distributed along the actinotrichia in control larvae at 2 and 3 dpf, which was arranged as a patterned fibrillar network of collagen that extended towards the edge of the fin (Fig 4A–4C). However, reduced fibrillar network and reduced Col2α1 expression were seen in the caudal fin of Elf3 morphants at 2 dpf (Fig 4B). At 3 dpf, moderately defective larvae had weaker Col2α1 expression in the actinotrichia compared to control, and the Col2α1 fibrils were disorganized (Fig 4C and 4D and insets). Severely defective larvae showed abnormal accumulation of Col2α1 with no fibrillary patterning in the caudal fin. Instead, high levels of poorly organized Col2α1 aggregates were present in the morphant (Fig 4E). Examination of Col2α1 expression in the median fin fold of control and Elf3 morphant revealed the presence of well-organized, thick Col2α1 fibrils in the control larvae. Severe disruption and disorganization of median fin fold collagen fibrils were seen in the Elf3 knockdown larvae (Fig 4F–4Q).

Elf3 morphants were shorter, bent, and had distorted/wavy notochords. The notochord functions as a hydrostatic skeleton in chordate embryos, rigid support provided by fluid pressure in the notochord vacuoles. Mechanical properties of notochord partially depend on its external fibrous matrix, which exerts the appropriate stiffness essential for lengthening of the embryo and maintenance of a straight axis. The notochord sheath cells produce the notochord fibrous matrix, which consist of Col2α1, other collagens, laminins, and other matrix molecules [62, 63]. The expression of Col2α1 was examined in the control and morphant embryos at 2 and 3 dpf. In the control embryo, Col2α1 was dispersed throughout the notochord in a uniform pattern at 2 dpf (Fig 4A, 4F and 4I). However, morphant embryos had disorganized Col2α1 expression patterns within the notochord. Moderately affected embryos had overall diminished Col2α1expression with fewer unevenly distributed higher Col2α1 expressing areas (Fig 4B, 4G and 4H). Severely affected embryos also had reduced Col2α1 expression in some areas and an accumulation of Col2α1 aggregates in other parts of the notochord (Fig 4B, 4G, 4H, 4J and 4K). The aggregates of Col2α1 were frequently seen in the bent notochord regions (Fig 4H–4K). At 3 dpf, control larvae had bands of high Col2α1 expressing areas in the anterior region of the notochord but not in the posterior region. Low levels of Col2α1 were evenly distributed between the bands and in the entire posterior region of the notochord of 3 dpf control larvae, which resembled the distribution of Col2α1 in 2 dpf embryos (Fig 4L–4O). In contrast, 3 dpf morphant larvae either did not have the segments of high Col2α1 expressing regions, rather showing low levels of collagen throughout the notochord (Fig 4M–4P) or irregularly spaced faint segments with very little or no Col2α1 expression in other parts of the notochord (Fig 4N–4Q). These observations confirmed that *elf3* gene function was required for proper ECM organization in the zebrafish fin and notochord, and Elf3 deficiency was associated with disorganization of the matrix.

### Elf3 deficiency disrupted optic nerve fasciculation and terminal axonal arborization in the optic tectum

**Fig 5:**
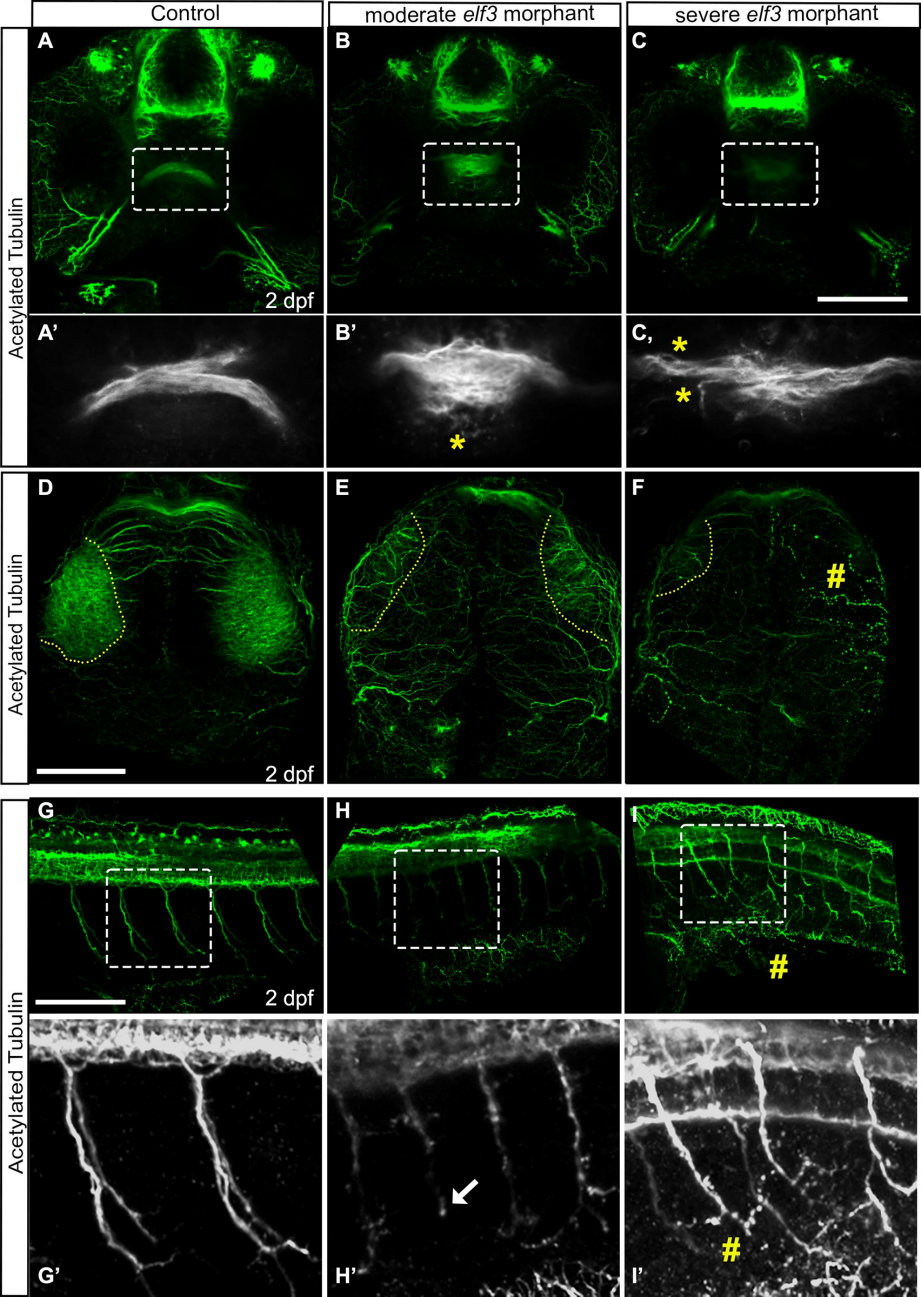
Elf3 deficiency caused optic nerve fasciculation and arborization in the optic tectum defects and fragmentation of spinal motor neurons. (A-C) Confocal imaging of acetylated tubulin stained embryos and 3D rendering shows tightly fasciculated optic nerves crossing at the optic chiasm in the control embryo (A) and a smaller de-fasciculated nerve in the morphants (B, C). (A’-C’) High magnification of the marked rectangle area in A-C. yellow asterisk show fibers de-fasciculated. (D-F) Confocal imaging of acetylated tubulin stained embryos and 3D rendering shows optic nerves arborized extensively within the tectal neuropil in the control embryo (D), and either fewer or no axon projections within that region in the Elf3 morphants (E, F). yellow perforated lines: presumptive tectal neuropil region; #: no axon projections. (G-I) 3D renderings of the confocal sections of acetylated tubulin stained 2 dpf control embryos showed thick bundles of caudal motor neurons arborized within muscle in control embryo (G, G’) and fragmented, abnormally branched and shorter axons in the morphants (H, H’, I, I’). (G’-I’) High magnification of the marked rectangle area in G-I. Arrow: short axon; #: fragmented axon. Scale bar for A-C, D-F and G-I = 100 μm.

Elf3 morphants exhibited delays in the eye cup formation and had smaller eyes. To examine the visual system of the morphants, acetylated-tubulin antibody labeling was performed that stains axons, including those in the optic nerve and optic tectum. Three-dimensional renderings of the confocal sections of the stained control embryos showed tightly fasciculated optic nerve projections of the retinal ganglion axons from each retina, which crossed at the optic chiasm and subsequently, projected toward the contralateral optic tectum (Fig 5A and 5A’). However, acetylated-tubulin stained optic nerves were smaller, the fibers were loosely packed and diffused in the morphants, indicating fasciculation defects. The defect was apparent near the optic chiasm where the axons defasciculated and spurious projections were clearly visible, indicating defects in midline pathfinding (Fig 5B, 5C, 5B’ and 5C’). Moreover, the optic chiasm in the Elf3 morphants were wider than the control embryos (Fig 5B and 5B’). Severely defective morphants displayed weaker acetylated-tubulin staining in the optic nerve, perhaps due to reduced ganglion cell numbers, but the staining in other nerves in the forebrain were not remarkably different from controls (Fig 5C and 5C’).

After crossing the midline, the optic nerves extend to the contralateral optic tectum in zebrafish where the nerve fibers ramified (branching) within the neuropil of the optic tectum, which are normally confined into the neuropil region. Acetylated-tubulin stained control embryos showed extensive arborization of optic nerve axons within the tectal neuropil (Fig 5D). However, Elf3 morphants had either few or no axon projections within that region, indicating that the optic nerves either did not extend into or had retracted from the tectum (Fig 5E and 5F). Axons may overshoot the optic tectum in the morphants (Fig 5E and 5F), suggesting possible axon pathfinding defects. Severely defective morphant embryos had reduced or degenerated axons.

### Deficiency of Elf3 changed spinal motor neuron morphology

In acetylated tubulin stained embryos, we noted defects in spinal motor neuron morphology in Elf3 morphants, indicating axonal growth and fasciculation defects. The morphology of neurons examined in 3D renderings of the confocal sections of acetylated tubulin stained 2 dpf control embryos showed thick bundles of caudal motor neurons that exited the spinal cord, spanned the entire ventral musculature, and arborized within muscle (Fig 5G and 5G’). However, Elf3 morphants had weakly stained spinal motor neurons that were fragmented or showed varicosities (swellings), and the axons were abnormally branched (Fig 5H, 5I, 5H’ and 5I’). Some of those axon projections were much shorter than normal (Fig 5H and 5H’). Measurements of the motor neurons in the anterior trunk showed that average axon lengths were reduced from 113.6 μm in controls (n = 18 neurons in 3 individuals, SD 14.65) to 68.3 μm in Elf3 morphants (n = 30 neurons in 4 individuals, SD 20.72), which was statistically significant (P<0.0001). Severely defective embryos showed more abnormal branching of shorter and fragmented axons (Fig 5I and 5I’). Taken together, Elf3 knockdown caused abnormal axonal pathfinding and axon degeneration in specific cell types.

### Elf3 functional interactions identified by pathway analysis

**Fig 6:**
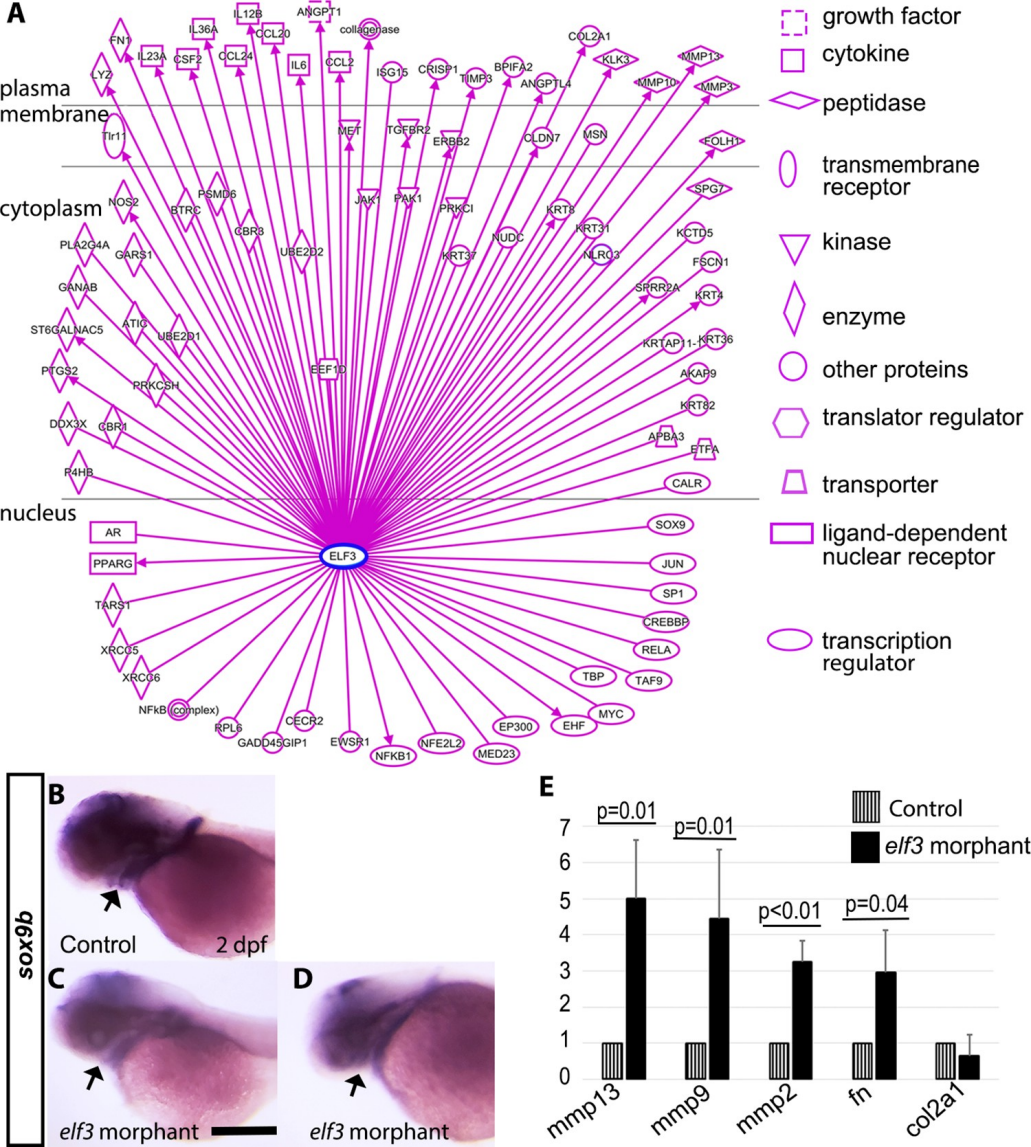
Dysregulation of extracellular matrix proteins in Elf3 morphants. (A) IPA predicted direct relationships and downstream targets of Elf3. (B-D) *In situ* hybridization detecting *sox9b* expression at 2 dpf show strong expression in the retina, brain ventricular zone, craniofacial cartilages, and pectoral fin in the control embryos (B) and weaker expression in those areas in the morphants (C, D). arrow: developing craniofacial cartilages. (E) Quantitative RT-PCR assays comparing transcripts of *mmp13*, *mmp9*, *mmp2*, *fn*, and *col2α1* (average fold change from at least 3 independent experiments) show altered levels of expression of those genes in morphants compared to control, Student’s t-test: P<0.05. Scale bar for B-D = 200 μm.

To identify potential molecular mechanisms for the developmental defects in Elf3 morphants, we performed IPA that predicts interactions of Elf3 with other molecules based on the manually curated content (from human, mouse, rat publications) in the Ingenuity Knowledge Base. IPA predicted 89 direct relationship (experimentally observed or highly predicted) and 89 nodes that are downstream of Elf3 (Fig 6A). Those molecules include transcription regulators, a translation regulator, a transmembrane receptor, peptidases, kinases, intracellular proteins, a growth factor, extracellular proteins, enzymes and cytokines. Out of fourteen IPA predicted transcription regulators, orthologous of thirteen of those (SOX9, JUN, SP1, CREBBP, RELA, TAF9, TBP, MYC, EP300, NFE2L2, MED23, EWSR1A, CALR) are expressed during zebrafish embryogenesis. To test how Elf3 knockdown affected the expression of these molecules, *in situ* hybridization and RT-PCR was performed. Sox9 plays multiple roles during zebrafish development, including cartilage, pectoral fin, eye, and neural crest development [64–66]. The Elf3 morphants showed defects in those tissues, which prompted us to analyze the expression of *sox9b*, one of the homologues of *sox9* by *in situ* hybridization. The probe detected *sox9b* in the retina, brain ventricular zone, craniofacial cartilages, and pectoral fin in the control embryos at 2 dpf (Fig 6B). Morphant embryos showed expression of *sox9b* in similar regions, but the intensity of the staining was lower than the controls indicating reduced *sox9b* expression (Fig 6C and 6D). Severely defective embryos showed little or no staining in the prospective branchial arch regions and very weak pectoral fin staining.

IPA indicated that Elf3 regulates the expression of many extracellular matrix proteins, including MMPs, Col2α1, and fibronectin. MMPs plays critical role in ECM remodeling and homeostasis and are essential for normal tissue development. Abnormal distribution of Col2α1 in various tissues in Elf3 morphants (Fig 4) supports this connection. Expression of Col2α1, other ECM molecules and ECM regulators (MMPs) were examined in Elf3 morphants. Quantitative PCR was done to measure the expression of transcripts for ECM proteins: Mmp13, Mmp9, Mmp2, Col2α1, and fibronectin. Elf3 knockdown significantly increased the levels of *mmp13*, *mmp9*, *mmp2*, *and fn* transcripts (~5 to 3 fold) in Elf3 morphants over control embryos at 2 dpf. The level of *col2α1* transcripts was minimally decreased (~0.7 fold) in Elf3 morphants as compared to control embryos, but this decrease was not significantly different from the control (Fig 6E). Taken together, Elf3 deficiency dysregulates the expression of extracellular matrix proteins, potentially causing ECM disorganization.

## Discussion

This study shows the importance of *elf3* during vertebrate embryogenesis and strengthens the findings of mouse knockout study that showed its requirement during mouse development [40]. Elf3 function was required for proper development of multiple organs and tissues in zebrafish.

Maternal *elf3* expression during epiboly was detected, but Elf3 knockdown did not affect this early developmental process. The role Elf3 on other early embryogenesis processes require additional studies. It is possible that early functions of Elf3 may be redundant.

High level of *elf3* mRNA was detected in specific regions of the developing central nervous system in zebrafish. Gene expression analyses during early human brain development also detected *ELF3* RNA in various brain segments (forebrain, midbrain, hindbrain, and spinal cord) of the mid-Carnegie stages embryos to fetus stages [67, 68]. The time of appearance and the RNA levels in brain regions was variable during human development. Elf3 knockdown affected brain morphology and produced a small eye phenotype in zebrafish. The morphants exhibited defects in the optic nerve fasciculation/arborization and spinal motor neuron projections. These observations indicate that Elf3 is required for proper neural morphogenesis. Additional studies focusing on Elf3’s role during neural development are needed to understand function.

The posterior axis did not develop normally in Elf3 morphants. Shorter anterior-posterior axis was also reported after ectopic expression of Ets-related gene (Erg) in *Xenopus* [69]. The distribution of Col2α1, a major component of ECM in notochord, was aberrant in Elf3 morphants. The vacuolar cells of notochord, which secrete Col2α, express *elf3*, indicating potential mechanism for notochord defect. For proper notochord morphogenesis, correct deposition and organization of the ECM are essential. Loss of *col8a1a* or *col1a1a* produced notochord defects in zebrafish similar to Elf3 knockdown [70, 71]. It is possible that notochord deformities and shortening of posterior axis in the Elf3 morphants was associated with disorganization of Col2α and other ECM components. Uneven distribution of Col2α1 in notochord and fins, showing areas with reduced expression and other areas containing aggregates, raises the possibility of ECM assembly/remodeling defects. A mouse study showed abnormally distributed and increased laminin-1 (a major ECM constituent of intestinal and connective tissues) staining in the small intestine and disorganization of the connective tissues underlying the villus lamina propria of the *Elf3*-deficient embryos [40]. Both mouse and zebrafish studies showed the role of Elf3 in cell-matrix interactions (see below).

Elf3 morphants showed craniofacial cartilage defects. Roles of other Ets family members in cartilage development were shown previously. Overexpression of Ets-related gene (Erg) in mouse sclerotome changed cellular morphology, reduced Alcian blue staining, decreased *Sox9* and inhibited chondrogenesis [72]. Increased dosage of Ets2 led to skeletal abnormalities similar to trisomy-16 mice and Down’s syndrome in human [73]. Another member, Ets1 regulates notochord formation [74]. Elf3-deficient zebrafish embryos had poorly developed branchial arches with fewer Col2α1 producing cells within those arches. Those embryos also displayed reduced *sox9b* expression. These observations, along with the missing pieces of neurocranium cartilages, suggest that the neurocranium defects in Elf3 morphants could arise early during chrondoprogenitor formation and migration. Presence of all branchial arches but reduced Alcian blue staining suggested near-normal initial steps but compromised chondrocyte differentiation step of branchial cartilage development.

Fin tissue architecture was defective in Elf3 morphants with frequent cysts or clumps of cells, which suggested problems in cell-cell and cell-matrix interactions. Altered fin organization was also reported after Mmp13 knockdown in zebrafish [22]. Hepatocyte growth factor activator inhibitor gene 1 *(hai1)* morphant exhibited lateral and caudal fin defects like the Elf3 morphant, which was shown to be associated to increased *mmp9* expression [75]. Knockdown of *mmp9* partially rescued fin defects of *hai1* morphant [75]. IPA identified that Elf3 interacts with multiple ECM proteins, including *mmp9* and *mmp13*, suggesting a regulatory role for Elf3 in ECM remodeling. Our experiments detected changes in ECM protein Col2α1 expression in fin. A dose-dependent requirement for Elf3 in maintaining the tissue architecture has been recognized.

MMPs play crucial roles during axonal growth and guidance [76]. Receptors in the axonal growth cones receive signals from their environment that direct axons to extend to their targets and arborize. MMPs modulate extracellular signaling pathways by selectively exposing cryptic ECM sites, inducing proteolysis of receptors and ligands [77, 78]. *Xenopus* studies showed that MMPs are important for both axon guidance and extension in the developing visual system [79, 80]. Inhibition of a broad-spectrum metalloproteinase by EDTA treatment reduced retinal axon ramification in the optic tectum of zebrafish [81]. Roles for MMP9 were reported for axonal outgrowth in the developing cerebellum of mice [82]. Paclitaxel is a widely used chemotherapy drug that also induces variable peripheral neuropathy in patients. A zebrafish study showed that Paclitaxel treatment induced axon degeneration in embryos and adult fish by ectopically inducing Mmp13 [83]. These studies underscored the importance of appropriate levels of MMPs for development and homeostasis. Our study showed upregulation of *mmp13*, *mmp9*, and *mmp2* after Elf3 knockdown. We speculate that optic nerve and motor neuron defects seen in Elf3 deficient embryos result from ECM remodeling and pathfinding signal recognition defects caused by MMP dysregulation, affecting axon projection and target recognition.

IPA identified interactions between Elf3 and many proteins belonging to various functional classes, which have been implicated in important developmental processes. Elf3 deficiency during development may directly or indirectly interferes with several signaling pathways and cellular processes, leading to the observed developmental defects. Our study showed that Elf3 is critical for embryonic development, and it is required for proper morphogenesis of the anterior-posterior body axis, craniofacial cartilages, fin tissue, optic nerve retinotectal projection and motor neuron pathfinding in zebrafish. Prior studies showed that the mouse knockout affected intestinal development, and Xenopus studies used gain-of-function, overexpression to show effects on axis development. Our findings show a set of new phenotypes that result from Elf3 loss-of-function and bioinformatics analysis points to potential pathways for gene activity. We hypothesize that these defects in Elf3 morphants are associated with ECM assembly and regulation, particularly collagen, producing defects in the craniofacial cartilage, notochord, and fins. Small brains and eyes were noted, and motor neuron projections were shorter and disorganized. In conclusion, our study showed that Elf3 is required for epidermal, mesenchymal, and neural tissue formation during development. Additional research is needed to fully understand the role of Elf3, a critical regulator of developmental signaling pathways, cellular processes, and cell-matrix interactions during development.

## Supporting information

S1 File (DOCX)
