# Supplementary material for: Elf3 deficiency during zebrafish development alters extracellular matrix organization and disrupts tissue morphogenesis
Source: PLoS One. 2022 Nov 16;17(11):e0276255. doi: 10.1371/journal.pone.0276255 (PMC9668168; doi:10.1371/journal.pone.0276255)

**Supplementary Material**

**Supplementary materials and methods**

**CRISPR/Cas9 targeting of *elf3* and analyses of injected larvae**

Independent validation of the elf3 morpholino oligonucleotide phenotype using CRISPR injection methods. Two gBlock gene fragments were ordered from IDT to make two guide RNAs (gRNA1: 5’ TGACTACTGCACCATGGAT 3’; and gRNA2: 5’ GAATCTGAACTACTGCACCA 3’). The gRNAs were made using MEGAscript™ SP6 Transcription Kit (Invitrogen AM1330) overnight at 37°C, followed by Ammonium acetate precipitation and subsequent EtOH precipitation, and redissolved in water. Less than 1,200 ng/µL gRNA at a 1:4 dilution of NEB nls-Cas9 protein (final concentration of 300-400 ng/µL gRNA) was injected into the 1-cell stage embryos. The injected embryos were raised until 5 dpf and analyses were done. Single larvae were imaged. The larvae then euthanized, transectioned, and the anterior portion was used for Alcian blue staining. Genomic DNA was extracted using the transectioned larval tail. Tissue was suspended in lysis buffer, boiled, cooled on ice, and digested at 55°C overnight with Proteinase K. The digested samples were boiled again and cooled on ice the next day followed by dilution in 0.1x Tris EDTA buffer. After genomic DNA preparation, PCR was run using 5 uL of the DNA and New England BioLabs Taq DNA Polymerase with Standard Taq Buffer protocol following manufactures protocol. Forward and Reverse primers were made and ordered from IDT (Forward: 5’ ACTAAACCTGAGCGGCTATTC 3’; Reverse: 5’ CTAGTGCTACACACAGGAGATG). PCR was analyzed by gel electrophoresis and purified following ThermoFisher Scientific ExoSAP-IT PCR Product Cleanup protocol. The purified PCR sample was sent to Eurofins Genomics with a sequencing primer from IDT for DNA sequencing (sequencing primer: 5’ CAGGTGAACCCGCAGATG 3’). DNA sequences were analyzed using SnapGene and NCBI Nucleotide BLAST to compare CRISPR/Cas9 injected embryo DNA to wildtype zebrafish DNA.

**Table S1: Sequences of the primers used, amplified transcript, and amplicon size.**

| **Primer** | **sequence** | **Amplified transcript** | **Amplicon size** |
| --- | --- | --- | --- |
| mmp13_F | 5’CCAGCGATGTGAGTCTGAG-3' | TCCAGCGATGTGAGTCTGAGGCTCAAGGAGATGCAGCAGTTTTTTAAGCTCAAGGTGTCTGGAAAGCTGGA | 71 |
| mmp13_R | 5'TCCAGCTTTCCAGACACCTT-3' |  |  |
| mmp9_F | 5'ATCTGTGTTCGTGACGTTTCC-3' | ATCTGTGTTCGTGACGTTTCCTGGAGATGTGATCAAGAACATGACCAACACACAGCTAGCGGATG | 65 |
| mmp9_R | 5'CATCCGCTAGCTGTGTGTTG-3' |  |  |
| mmp2_F | 5'GGCGAGTTCTGTAAGTTTCCA-3' | GGCGAGTTCTGTAAGTTTCCATTTTTGTTCATGGGTAAAGAGTACAACAGCTGCACCTCTCAGGGTCGAGATGA | 74 |
| mmp2_R | 5'TCATCTCGACCCTGAGAGGT-3' |  |  |
| fn1_F | 5'GGAGGGATCCTGTCTGACTG-3' | GGAGGGATCCTGTCTGACTGAAGCAGGTGTTTCCTATGCTCCAGGCATGCGCTGGAGCAAGGCTCAAGGTAGCAA | 75 |
| fn1_R | 5'TTGCTACCTTGAGCCTTGCT-3' |  |  |
| col2a1_F | 5'GAACTTCCTCAGGCTGCTGT-3' | GAACTTCCTCAGGCTGCTGTCTACTGAGGCCACTCAGACCATCACCTACCACTGCAAGAACAGCGTGGCTTACA | 74 |
| col2a1_R | 5'TGTAAGCCACGCTGTTCTTG-3' |  |  |
| rsp15_F | 5'CAGAGGTGTGGACCTGGACCAGC-3' | CAGAGGTGTGGACCTGGACCAGCTGCTGGACATGTCCTATGAGCAGCTGATGCAGCTGTATAGCGCCAGGCAGAGGAGGAGGCTGAACCGCGGCCTCAGGAGGAAGCAGCAGTCTCTCCTTAAACGCCTCCGCAAGGCCAAGAAGGAGGCGCCACCCATGGAGAAGCCAGAGGTGGTCAAAACTCACCTGAGAGACATGGTCATCCTGCCCG | 212 |
| rsp15_R | 5'CGGGCAGGATGACCATGTCTCTC-3' |  |  |

**Supplemental Figure Legends**

**Figure S1. Alignment of Zebrafish Elf3 and human ELF3 amino acid sequences.** ELF3 protein share 47.31% identity with human ELF3 protein but the ETS domain of zebrafish Elf3 shares 91.6% identity with human ELF3 ETS domain (blue highlighted region).


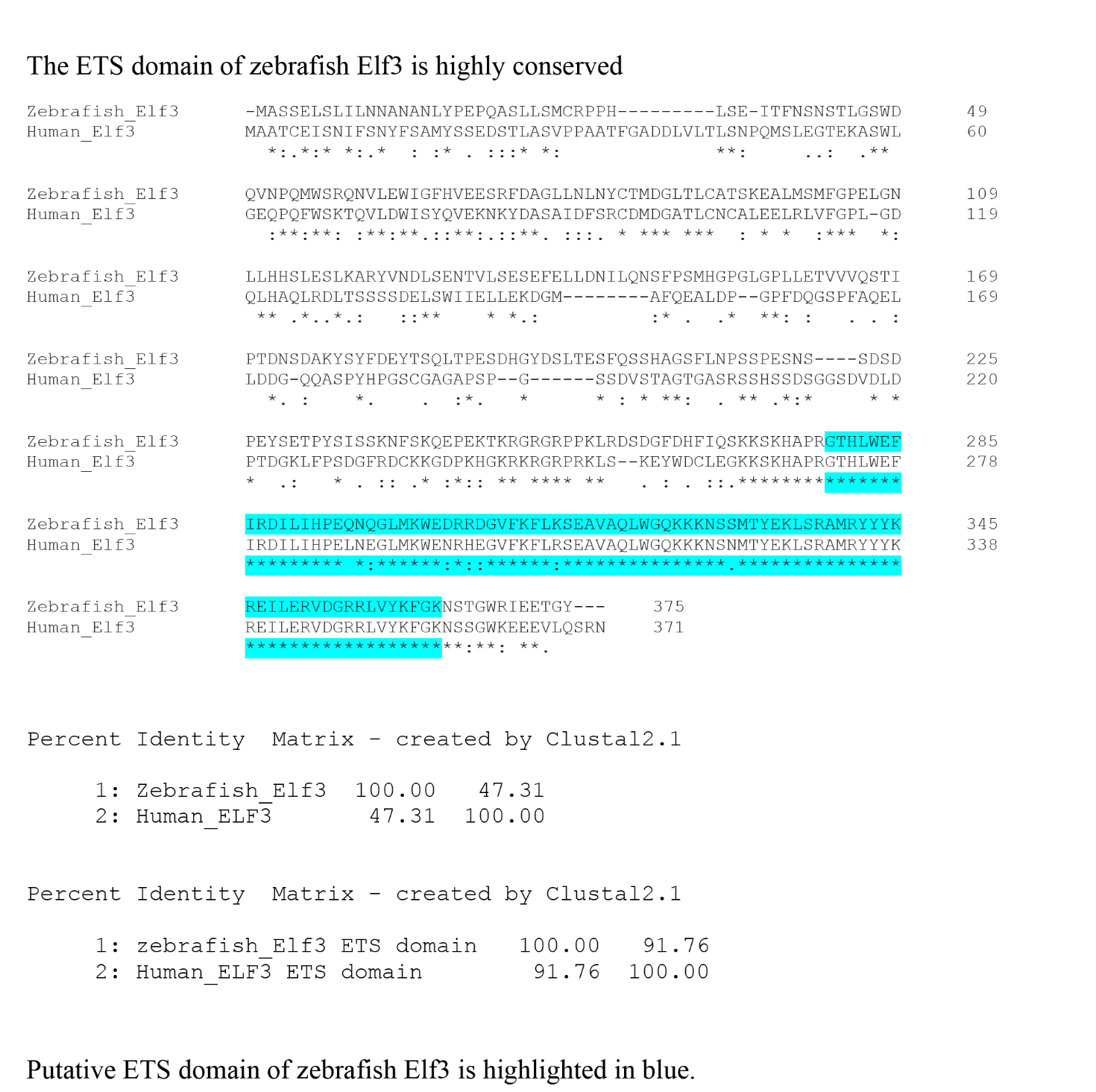


**Figure S2. Control morpholino injected embryos had no defects, and CRISPR/Cas9 *elf3* knockdown larvae phenocopy morpholino knockdown phenotype.** (A, B) Live images of 1 dpf uninjected control embryo (A) and control morpholino (1 nL of 0.1 mM) injected embryo (B). (C-G) Live images of 5 dpf uninjected control (C), gRNA1 injected (D, E) and gRNA2 (F, G) injected larvae. (C’, D’, E’, G’) Dorsal, lateral, and ventral views of Alcian blue stained head skeleton of control (C’) and gRNA injected (D’, E’, G’) larvae. (C”-G”) Electropherograms show the genomic DNA comprising *elf3* of the control larvae (C”; black lines on the electropherogram mark the gRNA1 and gRNA2 sequences) and the genomic DNA of gRNA1 (D”, E”) and gRNA2 (F”, G”) injected larvae. Red and blue highlights: CRISPR/Cas9 targeted regions of *elf3* and altered DNA sequences of the targeted regions in the injected larvae. Scale bar for A-B; C-G; and C’-E’, G’ = 200 µm.


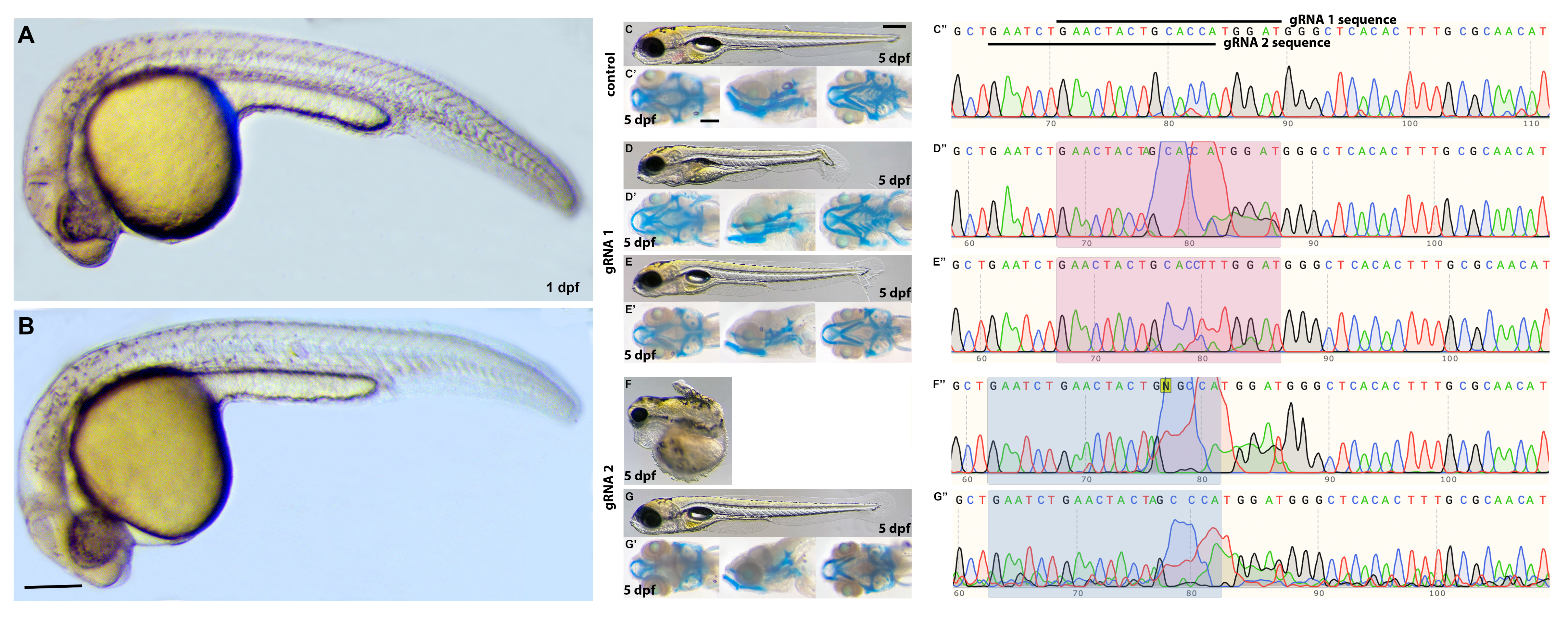


**Figure S3. Col2α1 stained embryos showed altered craniofacial cartilage development in Elf3 morphant.** (A-B”) Confocal Z-sections of Col2α1 stained craniofacial cartilages in the ventral (A, B), middle (A’, B’) and dorsal (A”, B”) positions of 3 dpf control larvae (A-A”) show developing Meckel’s, Ceratohyal, 1^st^ and 2^nd^ branchial arches, and ethmoid plate; Elf3 morphant larvae (B-B”) show a pair of cartilage (presumably Ceratohyal). (C-D”) Confocal Z-sections of E-cadherin (Cdh1) stained 3 dpf larvae show presence of E-cadherin in the developing cartilages of the control larva (C-C”), which is absent in the morphant larvae (D-D”). (E-F”) Merged images of Col2α1 and E-cadherin. Arrow points to the increased E-cadherin expression near the growing end of the cartilage. Scale bar for A-F; A’-F’; and A’’-F’’ = 100 µm.


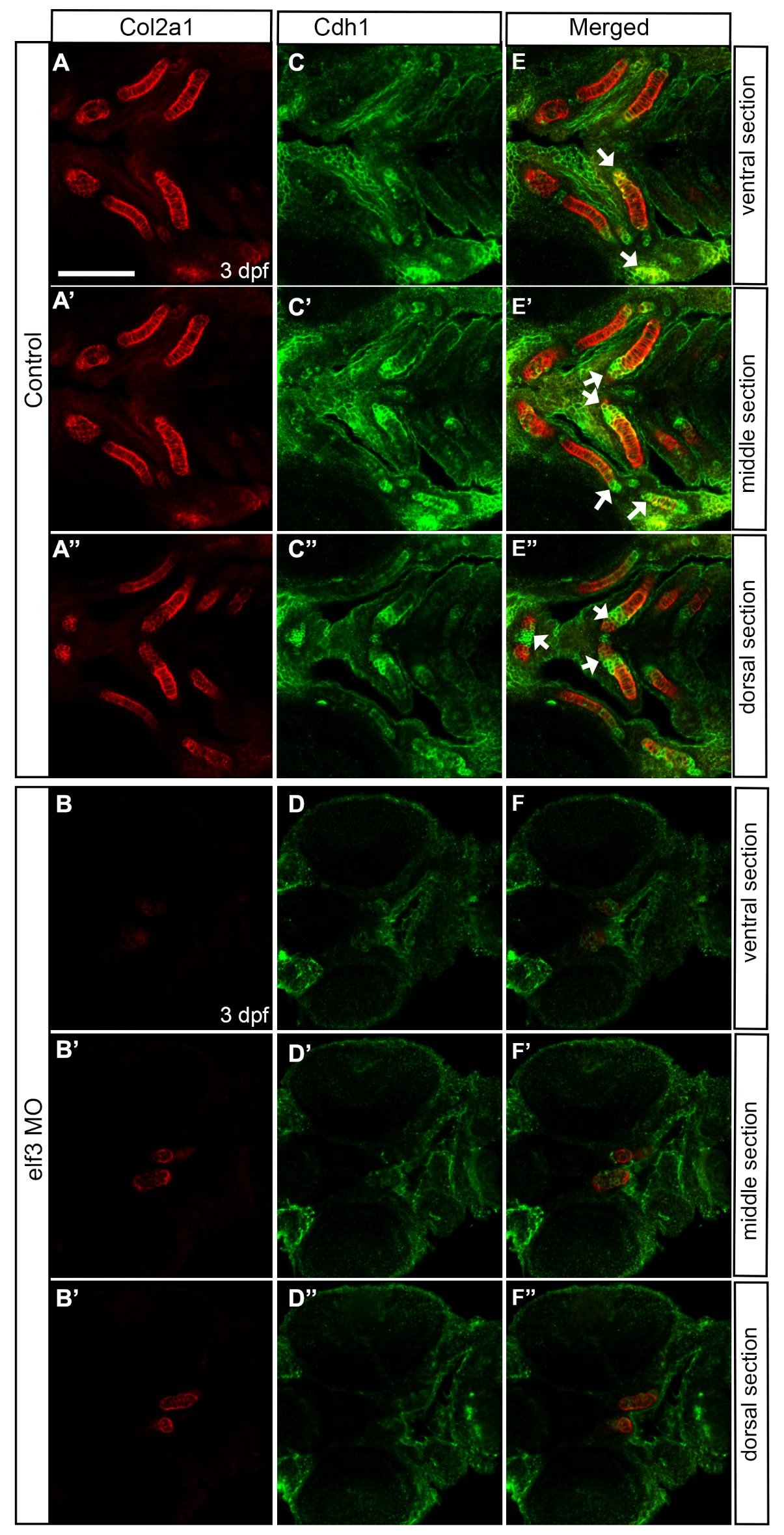


**Figure S4. Original, uncropped gel for Figure 1A.** Lanes 1 - 4 contains PCR DNA amplified from the cDNA made using oligo dT primer (Lane 1: 4.5 hpf; Lane 2: 8 hpf; Lane 3: 20 hpf; Lane 4: 24 hpf). Lane 5 - 8 contains PCR DNA amplified from the cDNA made using elf3 specific reverse primer (elf3-R primer) (Lane 5: 4.5 hpf; Lane 6: 8 hpf; Lane 7: 20 hpf; Lane 8: 24 hpf). Lane 9 contains PCR DNA amplified from the plasmid containing elf3 (pCBA3-zf-elf3). Lane 10 – 11 contain DNA ladders. To make the figure, lane 5 – 9 and 11 were cropped out, and only lane 1-4 and the marker lane 10 were used.


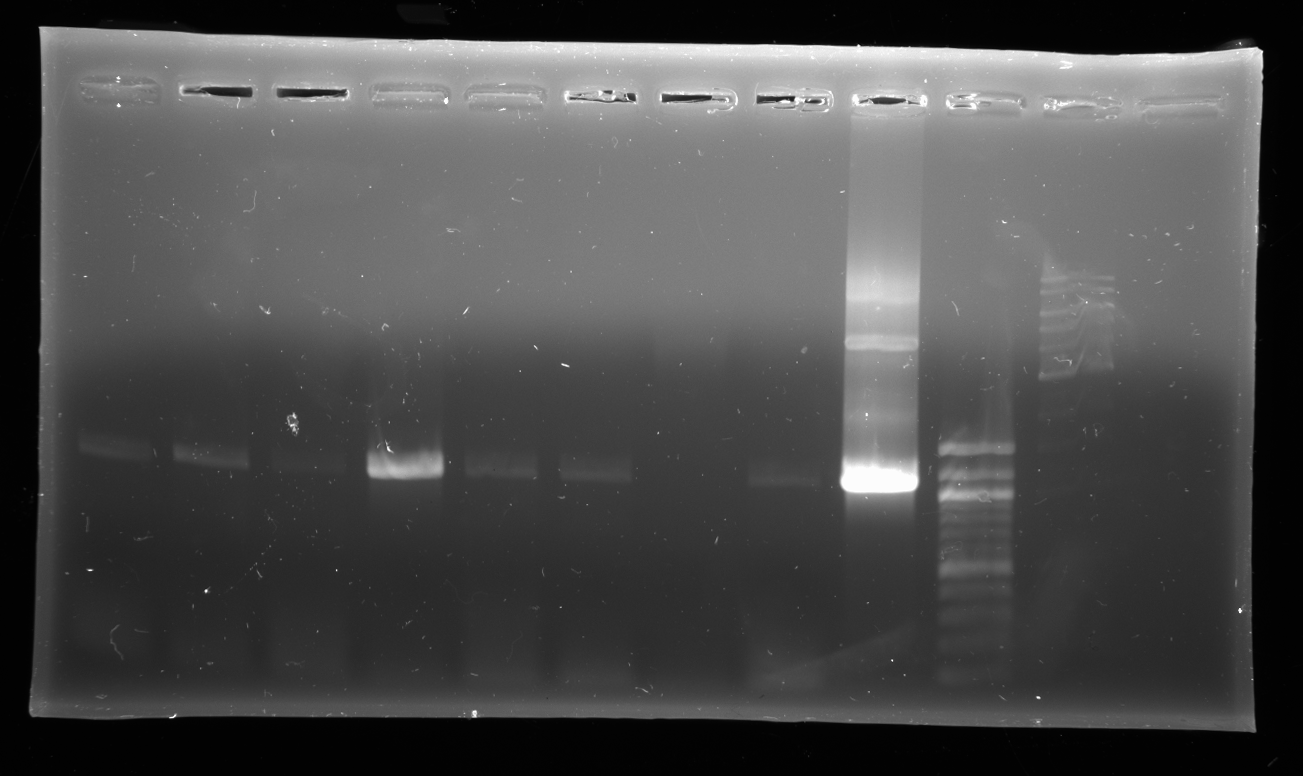

Supplement: S1 File — (DOCX) [file pone.0276255.s001.docx]
